# Transactions of the American Medical Association

**Published:** 1872-12

**Authors:** 


*The Transactions of the American Medical Association.* Vol. XXIII. Philadelphia: Printed for the Association, 1872. Buffalo: T. Butler & Son.

The meeting of the American Medical Association at Philadelphia was one which was full of interest, and many excellent papers were produced. The reports of the different sections are many of them full of interesting intelligence, and the papers published in connection with the reports are, in a majority of cases, thoughtful and scientific prodütions. The present volume contains the names of the officers and permanent members of the association since 1847, and an appendix containing a nomenclature of diseases.

